# Prolonged Pre‐Eruptive Phase of Herpes Zoster Ophthalmicus in Immunocompetent Athlete: A Case of Delayed Diagnosis and Management Challenges

**DOI:** 10.1002/ccr3.70399

**Published:** 2025-04-25

**Authors:** Sharanya Kumar, Rahul Tuli, Abdulsattar Mohammed, Brandon Duong, Kelly Downey

**Affiliations:** ^1^ Internal Medicine Riverside University Health System Medical Center Moreno Valley California USA; ^2^ School of Medicine University of California, Riverside Riverside California USA

**Keywords:** delayed diagnosis, headache, herpes zoster ophthalmicus, intractable pain, shingles, varicella‐zoster virus

## Abstract

Herpes zoster ophthalmicus should be considered in patients with intractable headache and eye pain, even without rash or classic risk factors. Early recognition of the pre‐eruptive phase and prompt antiviral therapy are crucial to prevent severe complications, including permanent vision loss or blindness.

## 1 Introduction

*Herpesviridae* is a family of DNA viruses, nine of which are known to infect humans [1]. Varicella‐zoster virus (VZV), alternatively known as human herpesvirus 3 (HHV‐3), is one of these viruses. Primary infections by VZV lead to chickenpox, which predominantly affects children without VZV immunity. Following primary infection, VZV exhibits neurotropism, allowing it to migrate to and establish latency within the dorsal root ganglia via retrograde axonal transport [2]. Reactivation of latent VZV within the dorsal ganglion can result in herpes zoster (HZ), colloquially known as shingles. This condition is often triggered by factors that weaken the immune system. Common risk factors for the development of shingles include age > 50, autoimmune diseases, and immunocompromised status, such as those infected with cancer or HIV. Shingles usually begins with a prodromal phase marked by viral symptoms such as fever, chills, malaise, and myalgias, followed by a characteristic vesicular rash distributed along a specific dermatome [3].

Herpes zoster ophthalmicus (HZO), which comprises 10%–20% of HZ cases, results from the reactivation of varicella‐zoster virus (VZV) in the ophthalmic branch of the trigeminal nerve (V1). This presentation is characterized by symptoms such as unilateral eye pain, tearing, headache, and vision loss. If left untreated, HZO may lead to permanent visual impairment [3]. In this report, we describe the case of an immunocompetent middle‐aged male presenting with a ten‐day history of intractable headache and unilateral eye pain, ultimately diagnosed with HZO.

## 2 Case History/Examination

A 45‐year‐old Caucasian male marathon runner with no significant medical history was transferred to the Emergency Department from an outside hospital due to a debilitating, intractable headache associated with stabbing left‐sided eye pain that began 7 days before. Associated symptoms included left‐eye photophobia, mild lacrimation, pain with rightward extraocular movements, and nasal congestion, without any evidence of conjunctival hyperemia or skin changes. Following admission, his headache intensity remained persistently severe, with occasional intermittent exacerbations lasting for an hour before returning to baseline. Despite administration of multiple analgesics, including morphine, dexamethasone, ketorolac, and fentanyl, his pain was unrelieved. His history was notable for a car accident one month before, resulting in residual neck pain, and a mechanical fall from a trampoline one week before admission, causing minor head trauma.

On physical examination, cranial nerves were intact, and sensation and strength were preserved bilaterally in both upper and lower extremities. Visual acuity testing revealed 20/20 vision in the right eye and 20/10 vision in the left eye, with an intraocular pressure of 12 mmHg in the left eye.

## 3 Methods (Differential Diagnosis, Investigations, and Treatment)

Initial diagnostic imaging, including non‐contrast CT head, CT orbits, and CTA of the head and neck, was unremarkable. Advanced imaging with MRI of the brain with and without contrast and MR venography also revealed no abnormalities. Lumbar puncture showed a mildly elevated opening pressure (27 cm H_2_O), no white blood cells, and slightly elevated glucose (77 mg/dL) and protein (50 mg/dL) levels. Cerebrospinal fluid cultures were negative for organisms. Symptoms persisted despite the lumbar puncture, and a dilated fundoscopic examination by ophthalmology ruled out papilledema. Neurology was subsequently consulted and suggested cluster headache or hemicrania continua as potential diagnoses.

The patient underwent multiple therapeutic trials of 100% FiO_2_ for half‐hour intervals without relief. A migraine cocktail comprised of IV valproate (1 g), prochlorperazine (10 mg), ketorolac (30 mg), dexamethasone (6 mg), and a one‐liter bolus of lactated Ringer's solution was trialed but was ineffectual. Escalating doses of gabapentin, indomethacin, acetaminophen, and intravenous hydromorphone pushes were then attempted but also failed to alleviate symptoms. Adjuvant treatments with intranasal lidocaine and ketamine infusion were similarly ineffective. Ultimately, the patient was placed on a patient‐controlled analgesia pump with IV hydromorphone, which provided transient pain relief but required frequent doses every 15 min.

**FIGURE 1:**
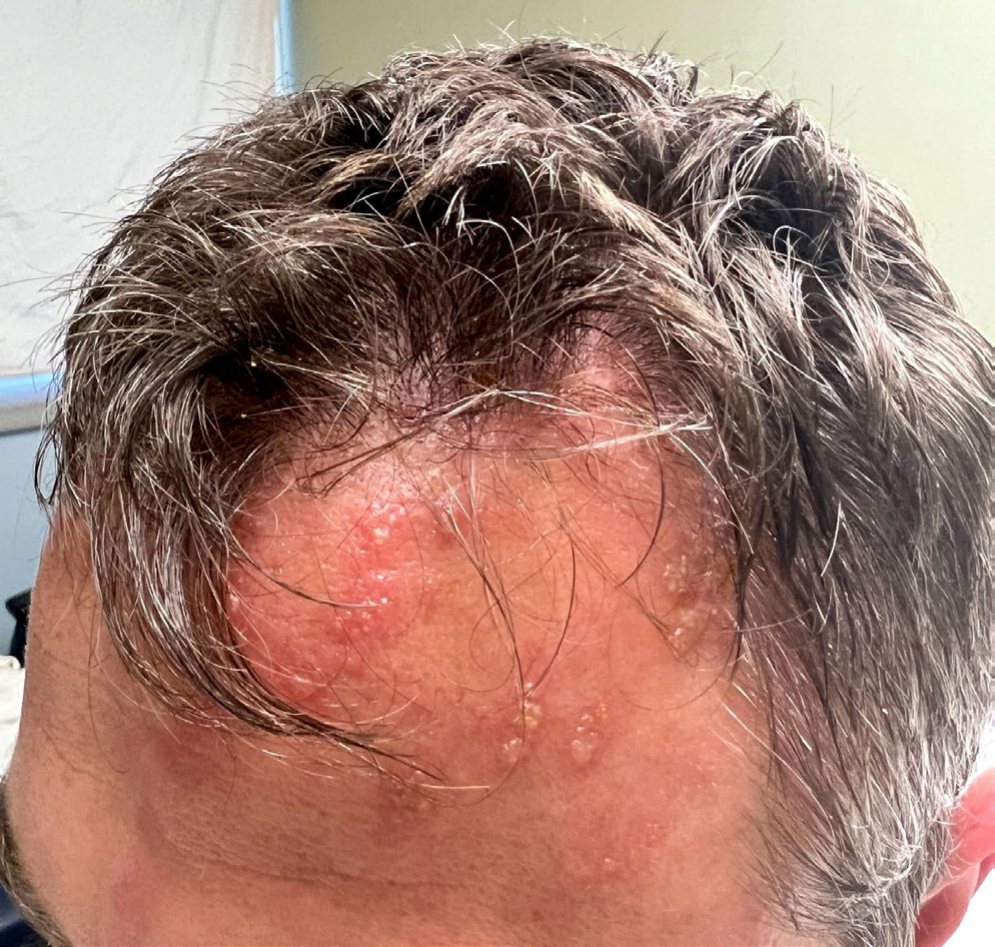
Vesicular dermatomal rash characteristic of herpes zoster ophthalmicus (HZO) involving the forehead and scalp, consistent with the distribution of the ophthalmic branch of the trigeminal nerve (V1).

Three days after admission, and 10 days from symptom onset, the patient developed an erythematous vesicular rash on the left forehead extending to the superior scalp, along with increased lacrimation and conjunctival erythema (Figure 1). Infectious disease consultation raised suspicion for HZO, which was supported by the patient's childhood history of Chickenpox. Varicella zoster serologies were ordered, and valacyclovir (1000 mg three times daily) was initiated.

## 4 Conclusion and Results (Outcome and Follow‐Up)

Ophthalmology re‐evaluated the patient and identified lesions on the left upper eyelid with palpebral conjunctival involvement, but no additional ocular pathology. Following initiation of antiviral therapy, the patient's symptoms rapidly improved, with a reduction in analgesic requirements. Serum studies revealed elevated varicella‐zoster IgM (1.45 ISR) and IgG (661.6 mIU/mL) levels, consistent with an acute active VZV infection. This was further supported by the patient's reports of increased work‐related stress and recent exposure to his sick daughter. Diagnostic workup for an underlying immunocompromised status was unremarkable, with negative results for syphilis, HIV, and hepatitis panels. The patient showed continued clinical improvement and was discharged with a 10‐day course of valacyclovir and an outpatient ophthalmology follow‐up in 2 weeks. At the time of discharge, opioid analgesia was no longer required.

## 5 Discussion

The varicella zoster virus is neurotropic in nature and resides within the sensory ganglion, with reactivation causing a painful and eruptive dermatomal rash. The virus inhibits multiple host defenses, which allows it to move along the sensory ganglion and establish latency. If viral replication continues to occur past the initial reactivation stage, then the sensory ganglion undergoes neuronal loss with the development of typical stabbing pain symptoms that occur along the ophthalmic distribution of the trigeminal nerve [4].

Approximately 75% of patients with HZO report pain as their initial symptom, typically followed by the appearance of the characteristic vesicular dermatomal rash within one to 7 days [3]. In this case, the pre‐eruptive phase was unusually prolonged, creating a significant diagnostic challenge as the sole presenting symptom was debilitating, intractable pain without a clear primary source and refractory to various analgesic modalities. Additionally, the patient lacked the typical risk factors that exist in patients who are diagnosed with HZO. Key differential diagnoses included cluster headache, hemicrania continua, and carotid artery dissection. The patient's lack of response to NSAIDs argued against a diagnosis of hemicrania continua, while the absence of improvement with supplemental oxygen made cluster headaches less likely [5, 6]. This expanded differential led to extensive imaging studies and pharmacologic interventions, resulting in a costly and protracted workup with unremarkable findings and a delayed diagnosis.

The patient's recent head and neck trauma further complicated the clinical picture. With the exceptional refractoriness to standard patient pain management regimens, an extensive differential diagnosis was formulated and investigated. The severity of symptoms initially raised suspicion for potential life‐threatening conditions such as vascular dissection or potential malignancy with cerebral compression; however, when these things were ruled out, the differential remained broad and unknown. This case exemplifies the need for clinicians to consider HZO in their differential when working up patients with intractable headache. Although this patient lacked the well‐established risk factors for development of shingles, it is still important to consider HZO because a delay or omission of diagnosis can lead to long‐term ocular ramifications such as loss of vision, glaucoma, and/or retinal necrosis [7]. One additional atypical presentation of HZ reactivation that should also be considered in a patient with neuropathic pain, but a lack of VZV‐associated pathognomonic rash, is zoster sine herpete (ZSH). ZSH is unique in that it can occur in a localized pattern, affecting the nerves directly and without spreading to the skin [8]. Life‐threatening conditions of ZSH include meningitis, encephalitis, cerebral vasculopathy, and death.

While the diagnostic process was prolonged due to the atypical presentation, managing the patient's pain proved equally challenging, necessitating exploration of multiple advanced therapeutic strategies. Although ineffective in this case, intranasal lidocaine can disrupt signaling at the sphenopalatine ganglion, blocking neuropeptides involved in the vasodilation that causes headaches [9]. Similarly, ketamine, an NMDA receptor antagonist, is gaining wide recognition for treating various pain‐related syndromes by blocking glutamate, a key molecule in pain perception [10]. Despite the lack of improvement in this patient, these interventions have shown value in the treatment of patients with complex pain syndromes [11, 12].

Despite these efforts, the persistence of symptoms underscored the critical importance of early recognition and prompt initiation of antiviral therapy in suspected cases of HZO or ZSH, as delays can result in severe and potentially irreversible complications such as stroke or blindness [3]. The most severe side effects of valacyclovir include kidney injury, confusion, anaphylaxis, and blood clots. For patients with comorbidities that could be exacerbated by one of these effects, a risk and benefit discussion should be initiated between the physician and patient to decide what is in the patient's best interest. However, if no contraindications are present and the clinical suspicion is high, it is recommended to start therapy immediately, ideally within 72 h of symptom onset [7].

This case underscores the necessity of timely diagnosis and preventive measures to mitigate the risk of severe complications associated with HZO. While therapeutic options remain critical, prevention and early clinical recognition form the cornerstone of optimal patient outcomes. The diagnosis of HZO is typically made through clinical examination alone, with additional lab tests generally unnecessary. However, if there is uncertainty about the etiology of a patient's presentation, testing for VZV IgG and IgM antibodies in serum or detecting VZV DNA via PCR can aid in confirming the diagnosis [13].

Detection of IgM antibodies via serology can indicate an acute infection, whereas elevated IgG antibodies can suggest a prior Chickenpox infection. PCR testing of a vesicle or cerebrospinal fluid sample can rapidly detect VZV DNA through DNA amplification, providing confirmatory evidence of an active infection. Both methods have their limitations. Serological testing may yield false‐negative IgM levels if performed too early in the infection, before the body has mounted a detectable immune response. Similarly, PCR's accuracy can be reduced if samples are taken from areas of low viral shedding, such as an underdeveloped or healing lesion. To improve diagnostic sensitivity, clinicians should consider performing both serological testing and PCR when evaluating patients suspected of having HZO [8, 14].

Currently, primary prevention consists of two doses of the varicella (chickenpox) vaccine during childhood, followed by two doses of the HZ (shingles) vaccine for adults older than 50 years of age [15]. If primary prevention is not possible, antiviral treatment is the mainstay of management, focusing on preventing complications rather than disease onset.

## Author Contributions

**Sharanya Kumar:** writing – original draft, writing – review and editing. **Rahul Tuli:** writing – original draft, writing – review and editing. **Abdulsattar Mohammed:** writing – review and editing. **Brandon Duong:** writing – review and editing. **Kelly Downey:** supervision, writing – review and editing.

## Ethics Statement

This study received an exempt determination from the institutional review board (IRB) of Riverside University Health System.

## Consent

The patient provided both verbal and written consent for the writing of this manuscript. All patient data has been properly deidentified prior to submission.

## Conflicts of Interest

The authors declare no conflicts of interest.

## Data Availability

All data generated or analyzed during this study are included in this article. Further enquiries can be directed to the corresponding author.
